# Guidelines for improvement of the procedural aspects of devices and surgical instruments in the operating theatre

**DOI:** 10.3389/fsurg.2023.1183950

**Published:** 2023-06-14

**Authors:** P. G. Calò, F. Catena, D. Corsaro, L. Costantini, F. Falez, B. Moretti, V. Parrinello, E. Romanini, A. Spinarelli, F. Venneri, G. Vaccaro, Scientific Technical Committee, G. Vaccaro

**Affiliations:** ^1^ Department of Surgical Sciences, University of Cagliari, Cagliari, Italy; ^2^ General Multi-Specialist Surgery, University Hospital of Cagliari, Cagliari, Italy; ^3^ Unit of Emergency Surgery, University Hospital of Parma, Parma, Italy; ^4^ International Research Department, BHAVE, Rome, Italy; ^5^ Department of Medical and Surgical Sciences, School of Community Medicine and Primary Care, University of Modena and Reggio Emilia, Reggio Emilia, Italy; ^6^ Multi-Specialist Department of Orthopaedics and Traumatology, Santo Spirito Hospital, Rome, Italy; ^7^ Multi-Specialist Department of Orthopaedics and Traumatology, Polyclinic University Hospital Consortium, Bari, Italy; ^8^ Quality and Clinical Risk Unit, University Hospital “G. Rodolico - San Marco”, Catania, Italy; ^9^ Guidelines Commission of the Italian Society of Orthopaedics and Traumatology, SIOT, Rome, Italy; ^10^ Clinical Risk Unit and Surgical Emergency, Florence Health Authority, Florence, Italy; ^11^ Social, Epidemiological and Outcome Research, BHAVE, Rome, Italy; ^12^ Education and Health Promotion, Catania Provincial Health Authority, Catania, Italy

**Keywords:** surgical site infections, SSI, surgical devices, perioperative setting, surgical instruments

## Abstract

Surgical site infections are a major complication for patients undergoing surgical treatment and a significant cause of mortality and morbidity. Many international guidelines suggest measures for the prevention of surgical site infections (SSI) in perioperative processes and the decontamination of surgical devices and instruments. This document proposes guidelines for improving the perioperative setting in view of the devices and instrumentation required for surgical procedures, aiming to reduce contamination rates and improve clinical performance and management for patients undergoing surgical treatment. This document is intended for doctors, nurses and other practitioners involved in operating theatre procedures, resource management and clinical risk assessment processes, and the procurement, organisation, sterilisation and reprocessing of surgical instruments.

## Introduction

Surgical site infections (SSIs) are a major complication for patients undergoing surgical treatment,representing a significant cause of mortality and morbidity, as well asincreased length of hospital stay, readmission rates, and hospitalization costs (1, 2). SSIs account for nearly 20% of all hospital-acquired infections in the United States (3). The type of procedure, the surgical technique, instrument reprocessing, and postoperative measures—all act as risk factors for SSI development,along withpatient-related determinants (4). Modifiable risk factors include the possibility of instrument contamination, the duration of the operation, the number of people present and the traffic in the room (5). Room air movement also seems to have a direct impact on the exposure of the surgical site to particles generated, exhaled or shed by patients and operators (6).

International guidelines suggest measures for the prevention of surgical site infections (SSI) in perioperative processes and the decontamination of surgical devices and instruments. Still, surgical instrument contamination is a major concern. Preoperative contamination seems largely dependent on the reprocessing procedures, whereas intraoperative contamination is affected by many different variables including airflow management, instrument handling, and times of exposure (4).

The operating theatre accounts for about 60% of overalloperational costs in the USA (7). Therefore, the economic implications of surgical procedure quality improvement are relevant. Improved efficiency in instrument reprocessing might significantly impact perioperative care costs and labor (8).

This document proposes guidelines for perioperative setting improvements given the devices and instrumentation required for surgical procedures, aiming to reduce contamination rates and improve clinical performance and management for patients undergoing surgical treatment.

To construct and disseminate useful recommendations on the use of disposable surgical procedure sets and the streamlining of conventional sets, a consensus process was adopted. This involved eight experts, including surgeons representing the Italian Society of Surgery (SIC), orthopaedic surgeons representing the Italian Society of Orthopaedics and Traumatology (SIOT) and a Clinical Risk Manager, and used the *Nominal Focus Group* technique of focused group discussion applied within the *Consensus Method*, and the *Delphi Technique* for the “nominal” phase with two rounds of assessment, thus combining a “real” phase of focused online group discussion with a pre-coded guide and an individual,precisely “nominal” phase. This was achieved by sending the material to be assessed online, along with a standardised questionnaireissued to determine the degree of agreement on the individual recommendations proposed.

The following questions were under consideration:

1) What is the relationship between the features of surgical procedure sets and the frequency of surgical site infections (SSI) in patients undergoing surgical treatment?
2) How do perioperative process times and operating theatre traffic vary in relation to the features of the procedure sets used?
3) What is the impact of streamlining and optimising surgical procedure sets on direct and indirect costs?

Some of the major findings and take-home messages from this work are the following:

• Procedural optimization appears to be a feasible way of addressing modifiable risk factors.
• Perioperative process times and operating theatre traffic can be affected by the procedure sets involved and can be reduced through set streamlining.
• Technological support for streamlining should be more considered both operationally and as future research

This document is intended for doctors, nurses and other practitioners involved in operating theatre procedures, resource management and clinical risk assessment processes, and the procurement, organisation, sterilisation and reprocessing of surgical instruments.

The structure and contents of this document derive from the work of the Advisory Board and an in-depth systematic literature review (9) and are written in accordance with the AGREE-IIChecklist (10).

## Policy and guidelines implications assessment

### Literature review

A systematic literature review (9) was conducted in accordance with the *Preferred Reporting Items for Systematic Review and Meta-Analyses—PRISMA* Statement 2020 (11) on the use and streamlining of disposable surgical procedure sets. The review was conducted blindly between authors and reviewers. The literature review process was carried out in the following stages:

1. Formulation of research questions by adapting the PICOS model
2. Processing of a search string applied to databases;
3. Collection of identified records;
4. Screening of records according to inclusion criteria;
5. Full-text selection of the studies identified during screening;
6. Data extraction from the studies included;
7. Thematic analysis of results

The search was applied to the following databases: MEDLINE, Embase, Web of Science, CINAHL, and The Cochrane Library.

The selection was made according to the following criteria:

• **Participants**: surgical specialists, operating theatre nurses and nurse coordinators, theatre technicians, sterilisation centre coordinators and operators, other personnel with clinical, organisational, or logistical roles; patients undergoing surgical treatment
• **Intervention:** use of conventional or disposable procedure sets; processes of streamlining surgical sets
• **Outcome**: incidence of SSIs, procedure time, in/out the traffic of personnel, costs related to surgical procedures
• **Setting**: general and specialist surgery facilities, supply services and sterilisation centres
• **Study designs**: qualitative, observational, nRCT and RCT, systematic reviews, guidelines. The studies selected were written in English and published until 28.12.2021.

Any previous guidelines, systematic reviews or meta-analyses discovered during the database search, concerning surgical sets from the point of view of clinical risk reduction, procedure optimisation and the streamlining of instruments, were selected and summarised in a separate table.

The main operational outcomes of interest were:

1. A reduction in the incidence of post-surgical SSIs;
2. A reduction in procedure times;
3. A reduction in operating theatre traffic flow;
4. A reduction in costs associated with the intervention.

### Collection and assessment of consensus

The degree of agreement among experts on good practices, and the level of consensus among the experts on the evaluation awarded to the good practices, were determined using the *Delphi Technique*.

### How to use the document

This document guides how to promote processes to improve devices and instruments with a view to improving performance and ensuring intra-operative safety for the patients and professionals involved. The information included should be used in conjunction with tools for reviewing evidence and developing evidence-based clinical practices. Althoughit is based on a literature review and the analysis of consensus among experts, fresh evidence and expert perspectives should be included in the decision-making process.

### Critical assessment of the strength of the recommendations

Each statement is presented with a classification of the level of evidence (Table 1) to facilitate a priority stratification of the good practices reported. The classification model used here was borrowed from other good practice documents from similar fields of study.

**Table 1:** Levels of evidence used in the present guidelines, based on the literature review results.

Category A: randomised controlled trials reporting comparative results for specific outcomes between different interventions
Level 1: meta-analysis of RCTs
Level 2: Multiple RCTs for which a quantitative summary could not be conducted.
Level 3: single RCT
Category B: observational studies or trials with no precise comparison groups, which could lead to an inference concerning the relationship between interventions and observed outcomes
Level 1: non-randomised comparative studies (quasi-experimental, cohort studies, case-control studies).
Level 2: non-comparative observational studies with measures of association (relative risk, correlation, sensitivity and specificity).
Level 3: non-comparative observational studies with descriptive measures (frequencies, proportions).
Level 4: case reports and case series.

The evidence to support the drafting of these guidelines was gathered through a systematic literature review. Additional evidence was gathered through a manual search and consultation with experts and board members. References to the results of the studies collected are provided with an indication of the category and level of evidence.

The categories of evidence (Table 1), labelled “Evidence” in the summary tables on good practice, represent the strength and quality of the design of the supporting studies. Each category is in turn subdivided into levels according to the strength and quality of the results obtained (significance, type of data, replication of results in different studies).

There was insufficient evidence when no studies that analysed a specified intervention according to an established outcome were collected, or when the studies analysed were not methodologically adequate. Occasionally, claims were formulated without the support of clear evidence but considered valid, particularly if borrowed from previous guidelines, even if there were no precise bibliographical references. In such cases, the level of evidence is classified as “not defined”.

### Degree of uncertainty

Following a standardised assessment of the risk of bias in the individual publications, the degree of uncertainty is expressed as a judgement on the overall risk of bias, labelled as “Uncertainty” in the summary tables on good practice, and “high”, “unclear”, “low” or “not defined” in the individual statements. The tools used to assess the risk of bias and the results of each study are stated in the literature reviews.

### Risk of bias in the studies included in the review

The quality of the original studies included was assessed using the following tools: RoB-2 (12) for randomised controlled trials; QUADAS-2 (13) for diagnostic accuracy studies; ROBINS-I (14) for observational studies; ROBIS (15) for systematic reviews.

### Opinions

The statements were also classified based on the opinions of the experts and board members and represented by an O (“Opinion”), taking into account both the degree of agreement and the level of consensus expressed by the group of experts on the specific claim.

### Degree of agreement

The degree of agreement with the claims among the experts, labelled “Agreement” in the summary tables, was represented as a central trend index (mean value) of the scores the experts assigned to the claims on a scale of 1 to 5, where 1 represents the minimum level of agreement with the claim and 5 the maximum level. The degree of agreement is a measure of *how much* the panel “approved” the claim.

### Level of consensus

The level of consensus among the experts on the assessment given to the claims, labelled “Consensus” in the summary tables, is represented using a classification in six categories: Unanimity, High, Medium-High, Medium, Medium-Low, and Low. The reference (with cut-offs to the classes indicated of 0; 0.5; 0.8; 0.9; 1, respectively) is to the values of a score dispersion index (standard deviations). This indicates how uniform or non-uniform the opinion of the panel was.

## Actionable recommendations

### Clinical impact of the choice of surgical devices and instruments

**Claim 1.** The use of disposable procedure sets can reduce the incidence of surgical site infections and the associated consequences. (Evidence: B1. Uncertainty: unclear. Agreement: 4.5. Consensus: Medium-High).

Despite the progress made, the incidence of contamination of surgical devices and instruments is still significant (4) The decision to use disposable devices and instruments has been accompanied by a lower incidence of surgical site infections (16) and surgical revisions for remediation (17).

**Claim 2.** The use of a separate surgical set to close the wound helps reduce the incidence *of SSIs. (*Evidence: B1. Uncertainty: unclear. Agreement: 4. Consensus: Medium-High).

An SSI prevention bundle, trialled on 233 patients with a statistically significant reduction in the incidence of infections, included a separate surgical set for the final packing of the surgical wound (17).

### Procedures for streamlining surgical sets

**Claim 3.** Streamlining surgical sets is widely validated in many contexts of general and specialist surgery. (Evidence: B3. Uncertainty: unclear. Agreement: 4.1. Consensus: Medium).

The studies presented in the literature involved multiple branches, including orthopaedics (*n* = 6), gynaecology (*n* = 4), ENT (*n* = 6), thoracic surgery, endocrine surgery, paediatric surgery (*n* = 2), neurosurgery, ophthalmology, vascular surgery, breast surgery (*n* = 2), urology, general surgery (*n* = 2), hand surgery, and plastic surgery.

**Claim 4.** It is recommended that the streamlining of surgical sets begins with a phase of direct or indirect observation, possibly supported by computational systems, to highlight the usage status of devices and surgical instruments from a qualitative and quantitative point of view. (Evidence: B3. Uncertainty: unclear. Agreement: 4.4. Consensus: High).

Several studies included an observation phase involving the preparation, use and reprocessing of the surgical sets between the operating theatre and the sterilisation centre (*n* = 13).

The extent to which an instrument is used, and therefore the benefits of keeping it in the set or not, was assessed on an objective basis by analysing the data collected during the observation (*n* = 13), with the cut-off of use generally considered to be 20% to 25%. In other cases, the selection was made on a subjective basis by professionals (*n* = 3), by consensus or by the collection of questionnaires on perceived use. In one case a heuristic mathematical model was developed based on a discussion with skilled surgeons on their preferences as to what is included in the individual sets (18).

**Claim 5.** The streamlining procedure can be introduced through staff training on the rationale, objectives and meaning of the procedure. (Evidence: B3. Uncertainty: unclear. Agreement: 4.8. Consensus: High).

Training and awareness-raising, with the active involvement of all stakeholders, appear to play a decisive role in the adherence of practitioners to streamlining practices and the long-term maintenance of the results obtained (19).

**Claim 6**. *Assessment of the usage status of surgical devices and instruments can be improved, though not replaced, by the subjective contribution of the practitioners involved, through multidisciplinary discussion or the administration of ad hoc questionnaires. (*Evidence: B3. Uncertainty: unclear. Agreement: 4.5. Consensus: Medium-High).

One study analysed the difference between the perceived use of surgical devices and instruments by the surgeons involved and their actual use determined by observation. The results were 37% and 55% (20) respectively. It is therefore believed that, in the context of streamlining devices and surgical instruments, a subjective assessment may increase the clinical risk caused by underestimation of the use of a given device or instrument.

**Claim 7.** Depending on the observed extent to which they are used, lesser-used devices and surgical instruments can be removed from the surgical set. A utilisation cut-off of 20% to 25% is sufficient to achieve satisfactory streamlining. (Evidence: B3. Uncertainty: unclear. Agreement: 4.3. Consensus: High).

Generally, the studies identified considered the removal of the surgical devices and instruments used in fewer than 20%–25% of cases. One study reported a comparison between the extent of utilisation before and after the optimisation of two sets, showing an increase in use of 27% to 30% (21).

**Claim 8.** Streamlined sets should be reviewed by a multidisciplinary working group before implementation, and any devices and instruments considered necessary must be returned. (Evidence: B3*.* Uncertainty: unclear*.* Agreement: 4.8*.* Consensus: High).

In some cases, only surgeons were involved (*n* = 6). In other cases, the formation of a multidisciplinary team was encouraged (*n* = 7). In most of the studies, the new optimised set was presented to the clinicians and reviewed. Devices or instruments were then added according to their opinion.

**Claim 9**. *Devices or instruments that are not being used must be packaged in a separate set, to be available when needed. (*Evidence: B3. Uncertainty: unclear. Agreement: 4.6. Consensus: High).

Surgical devices or instruments that were excluded were generally packaged in a dedicated set, or the original set remained available. In seven studies the frequency of use of the devices or instruments excluded was measured as a marker of the safety of streamlining over a pre-specified period. Considering that a total of eight sets were optimised, the frequency was 0% (*n* = 5), 0.9%, 6% and 10% respectively. The fact that cases have been observed where an excluded surgical device or instrument was needed implies that, in the interests of minimising clinical risk, devices or instruments that are conventionally present must be available to the team when needed.

**Claim 10**. Observation after the implementation phase should examine the efficacy and safety outcomes of the streamlining procedure. The time taken to prepare the operating theatre, the exposure of devices and surgical instruments to the air for the entire duration of the surgery, the number of staff present and the flow of traffic through the room, can be used as surrogate outcomes for the contamination of devices, instruments and the surgical site. (Evidence: B3. Uncertainty: unclear. Agreement: 4.8. Consensus: High).

Eleven studies measured the time taken to set up the operating theatre, which decreased from 2 to 5 min following the implementation of optimised sets. Other studies observed that optimisation of the set had an impact on the overall duration of the procedure, with a 5 to 6-minute reduction. The duration of surgery is a confirmed risk factor in the occurrence of SSIs (22). A reduction in the time spent cleaning the operating theatre (−25%, *n* = 1), and the time spent by the nurse on duty outside the theatre for reasons related to the retrieval of surgical devices and instruments (−15.5%, *n* = 1), were also observed. One study observed a downtime of 9% of the entire procedure due to reasons related to the retrieval of surgical devices and instruments (23). One study measured a reduction in the frequency of procedure cancellations following the implementation of an optimised set: incidents dropped from 3.9% to 0.2% (8).

## Discussion

This document provides indications on how to encourage optimization processes for devices and instruments to improve performance and ensure the intraoperative safety of the patient and the professionals involved. An attempt has been made to answer both clinical and organizational questions that seemdeeply intertwined.

While the available evidence allows low levels of certainty -and the roles of bias have often been unclear—we remark on the high level of accordance in the Advisory Board on the claims proposed (Table 2).

**Table 2:** Summary of claims.

Claim	Evidence	Uncertainty	Agreement	Consensus
*1. The use of disposable procedure sets can reduce the incidence of surgical site infections and the associated consequences.*	B1	Unclear	4.5	Medium-High
*2. The use of a separate surgical set to close the wound helps reduce the incidence of SSIs.*	B1	Unclear	4	Medium-High
*3. Streamlining surgical sets is widely validated in many contexts of general and specialist surgery.*	B3	Unclear	4.1	Medium
*4. It is recommended that the streamlining of surgical sets begins with a phase of direct or indirect observation, possibly supported by computational systems, to highlight the usage status of devices and surgical instruments from a qualitative and quantitative point of view.*	B3	Unclear	4.4	High
*5. The streamlining procedure can be introduced through staff training on the rationale, objectives and meaning of the procedure.*	B3	Unclear	4.8	High
6. *Assessment of the usage status of surgical devices and instruments can be improved, though not replaced, by the subjective contribution of the practitioners involved, through multidisciplinary discussion or the administration of ad hoc questionnaires.*	B3	Unclear	4.5	Medium-High
*7. Depending on the observed extent to which they are used, lesser-used devices and surgical instruments can be removed from the surgical set. A utilisation cut-off of 20% to 25% is sufficient to achieve satisfactory streamlining.*	B3	Unclear	4.3	High
8. *Streamlined sets should be reviewed by a multidisciplinary working group before implementation, and any devices and instruments considered necessary must be returned.*	B3	Unclear	4.8	High
*9. Devices or instruments that are not being used must be packaged in a separate set, to be available when needed.*	B3	Unclear	4.6	High
*10. Observation after the implementation phase should examine the efficacy and safety outcomes of the streamlining procedure. The time taken to prepare the operating theatre, the exposure of devices and surgical instruments to the air for the entire duration of the surgery, the number of staff present and the flow of traffic through the room, can be used as surrogate outcomes for the contamination of devices, instruments, and the surgical site.*	B3	Unclear	4.8	High

As remarked by other authors, conclusive evidence on the relation between SSIs and surgical instrumentation is still lacking (4). Nevertheless, procedural optimization appears to be a feasible way of addressing modifiable risk factors. Perioperative process times and operating theatre traffic can be affected by the procedure sets involved (4, 9) and can be addressed through set streamlining.A wide part of our guidelines regards the optimization of surgical instrumentation. The literature review purposefully considered many quality improvement reportsto catch the features that future programs should showcase. We have stressed the importance of observation and multi-professional engagement in optimization processes. We have called for structured methods of leading change programs as we are aware that finding solutions is not enough: quality improvement greatly depends on handling human and relational factors (8). Additionally, we remark that solutions need to be proved acceptable from an economical perspective, given the impact of perioperative procedures on the health care budget.

Many questions remain for future research. First, surgical set streamlining still needs supporting evidence to be related to instrument contamination rates in theoperating theatre. Second, the influence of patient-related factors on the use of surgical instruments and perioperative times should be studied to improve risk assessment and programcounteractions. Finally, technological support forstreamlining should be more considered.

## Data Availability

The original contributions presented in the study are included in the article, further inquiries can be directed to the corresponding author.

## Group members of the Scientific Technical Committee

G. Vaccaro; PhD, Methodologist, Director of Social, Epidemiological and Outcome Research, Methodological Advisor, Bhave; Sociologist, Education and Health Promotion Unit, Catania Provincial Health Authority. L. Costantini; MD, School of Community Medicine and Primary Care, Department of Medical and Surgical Sciences, University of Modena and Reggio Emilia—Italy. D. Corsaro; Bhave International Research Manager, BHAVE –Rome, Italy.
